# A comparison of supracostal and infracostal access approaches in treating renal and upper ureteral stones using MPCNL with the aid of a patented system

**DOI:** 10.1186/s12894-015-0097-3

**Published:** 2015-10-13

**Authors:** Difu Fan, Leming Song, Donghua Xie, Min Hu, Zuofeng Peng, Xiaohui Liao, Tairong Liu, Chuance Du, Lunfeng Zhu, Lei Yao, Jianrong Huang, Zhongsheng Yang, Shulin Guo, Wen Qin, Jiuqing Zhong, Zhangqun Ye

**Affiliations:** Department of Urology, The Affiliated Ganzhou City People’s Hospital of Nanchang University, Ganzhou, Jiangxi 341000 China; Dermatology Institute of Gan County, Jiangxi, 341100 China; Department of Urology, Tongji Hospital, Tongji Medical College, Huazhong University of Science and Technology, Wuhan, Hubei 430030 China

**Keywords:** Patented system, Percutaneous nephrolithotomy, Percutaneous tract access

## Abstract

**Background:**

There are still disagreements on which is a better approach to choose to establish percutaneous tract for percutaneous nephrolitotomy (PCNL), between supracostal and infracostal approaches. The aim of this study is to investigate the safety, efficacy and practicability of minimally invasive PCNL (MPCNL) with the aid of a patented system either through supracostal or through infracostal access.

**Methods:**

A retrospective study was carried out for 83 patients with renal or upper ureteral stones. Under the guidance of B ultrasound or C-arm, these patients were treated by MPCNL through either 12th rib infracostal (Group 1, 43 cases) or supracostal (Group 2, 40 cases) access approach. These 2 groups were compared for total number of percutaneous tracts, average time in establishing a given percutaneous tract, the number of percutaneous tract used for each case, the average stone clearance time, the clearance rate of all stones by one surgery, and the amount of bleeding using a single percutaneous tract.

**Results:**

There was a significantly smaller total number of percutaneous tracts needed, a smaller number of cases that needed two percutaneous tracts to clear stones completely, a shorter average time in establishing a percutaneous tract, and a smaller average amount of bleeding in infracostal access group. At the same time, there were a significantly larger number of cases in which stones were cleared completely using a single percutaneous tract and a higher renal stone clearance rate by one surgery.

**Conclusion:**

There were several advantages of infracostal access. These included accuracy in establishing a percutaneous tract, safety, quickness, convenience and flexibility in moving the patented sheath, and higher renal and upper ureteral stone clearance rate by one surgery.

## Background

Since some scholars [1] first proposed a minimally invasive percutaneous nephrolithotomy (MPCNL) to remove stones, MPCNL has gradually been accepted by many patients and urologists in China, as a significant improvement over standard percutaneous nephrolithotomy (PCNL), and has become one of the most important ways to treat urinary tract stones in China [2–4].

To perform PCNL smoothly and successfully, it is critical to choose a suitable percutaneous tract access approach. A small-sized and highly efficient percutaneous tract is desirable to avoid injury from the puncture and percutaneous tract establishment, and to reduce complications [5]. Currently, most scholars agreed that PCNL through a supracostal access approach can clear stones efficiently with a low rate of complications in treating staghorn renal calculi and upper ureteral stones [6]. From August 2008 to April 2011, 83 patients who met the group inclusion standard underwent the MPCNL with 43 cases of renal stones and 40 cases of ureteral stones either through a 12th rib infracostal access approach (Group 1, 43 cases) or a 10^th^ to 12^th^ rib supracostal access approach (Group 2, 40 cases). All these patients were treated by MPCNL with the aid of a patented stone-breaking and clearance system (patent number ZL200820137434.6). We compared these 2 groups on different markers and reported the results as below.

### Patients and Methods

First of all, the study was performed with the approval of ethics committee at the Affiliated Ganzhou City People’s Hospital of Nanchang University, China, in compliance with the Helsinki Declaration. Written informed consent was obtained from every patient for publication of this research report and any accompanying images.

## Methods

A retrospective study was carried out for the 83 patients with renal or upper ureteral stones. The inclusion standards were as below. Complete or incomplete staghorn renal stones with sizes ≥2.0 cm; renal calyceal stones with co-existing calyceal obstruction and clinical symptoms; ureteral stones above L4 and >1 cm in size, stone has stayed in the ureter for >2 months complicated by ureteral ectasis above the stone, or patients who have previously failed shock wave lithotripsy (SWL) or ureteroscopic lithotripsy. Some of the patients who had anemia which was rectified before inclusion in the study. All patients with hypertension, diabetes, abnormal heart and lung function, or patients who were too obese to tolerate a prone position during surgery were excluded. The stone burden (cm^2^) was calculated by multiplying maximal length and maximal width of the stone in plain film of kidney-ureter-bladder (KUB).

Surgery took place under continued epidural anesthesia or general anesthesia. The patient was first placed in a lithotomic position and then a prone position. The abdomen was not boosted, as previously described [7].

**Fig. 1:**
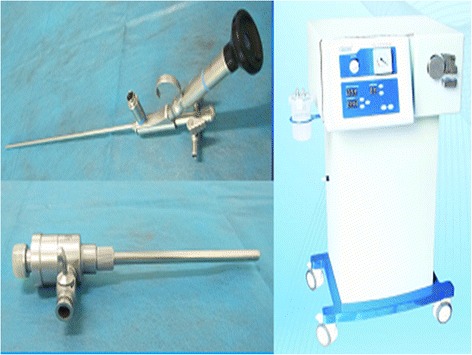
Lithotripsy and suctioning/clearance system comprised of small diameter percutaneous nephroscope, patented sheath, and irrigation and suctioning system

Real time ultrasonography or C-arm-guided percutaneous punctures were made with an 18-gauge coaxial needle into the targeted calyx. When using an infracostal access approach the puncture point was located below the 12^th^ rib or at the tip of the 12^th^ rib between the posterior line axillary and linea scapularis. The targeted point was one of the rear group calyces in the lower or middle pole. When using supracostal access approach the puncture point was located between the 11 th rib supracostal and the 12th rib supracostal. The targeted point was one of the rear group calyces in the upper or middle pole. For both access approaches, we always minimized the angle between the long axis of our percutaneous tract and the long axis of the collecting system when we were dealing with renal staghorn stones. The point we preferred to target was the calyx with the stone inside that was closest to our puncturing point. When we were dealing with ureteral stones we always minimized the angle between the long axis of our percutaneous tract and the axis between the proximal to the distal segments of the ureter. The puncture point was in the 11th intercostal space or the 12th subcostal margin, between the posterior axillary line and scapula line. The puncture was judged successful if there was urine overflow or if it touched a stone. Zebra guidewire was inserted and fixed. The puncturing needle was then taken out. After a 0.5–0.7 cm skin incision, the dilatation of the percutaneous tract was performed serially over the guidewire with a fascial dilator to 16 F. A 16 F patented sheath (Fig. 1) was placed at the percutaneous access port and was connected to a vacuum aspiration machine, as previously described [7]. Subsequently, a small diameter nephroscope (12Fr) was inserted through the sheath to explore stones. A holmium laser was used to break the stones and a vacuum suctioning device was used to clear gravel, as previously described [7]. When residual stones found using intraoperative real-time ultrasonography or C-arm needed a second or third percutaneous tract to clear stones, the 2^nd^ and/or 3rd percutaneous tract(s) was/were established using an infracostal approach to target one of the rear group calyces in the lower or middle pole. The amount of intraoperative bleeding was calculated using a hydrogenated high iron hemoglobin method, as previously described [7, 8]. A KUB was taken 3 to 5 days after surgery, and a computerized tomography (CT) was performed for cases with uric acid stones, to check for residual stones. If no residual stones > 4 mm were present, which was defined as stone-free, the nephrostomy tube was removed and no further treatment was pursued. Otherwise, a second-stage percutaneous nephrolithotomy or SWL treatment was performed, as previously described [7]. Total percutaneous tract number, average time in establishing the percutaneous tract, the number of percutaneous tract used for each case, the average stone clearance time (From the beginning of stone clearance to the end of the nephrostomy tube indwelling), one time stone clearance rate, one stage renal stone clearance rate and bleeding amount using a single percutaneous tract were recorded as data. Complications were evaluated according to the Clavien classification.

### Statistics

All data were analyzed using SPSS11.5. A Student’s *t*-test was used for quantitative variables and a Chi-square test was used for qualitative variables. *p* < 0.05 was used to indicate statistical significance.

## Results

There were 43 cases of renal stones (staghorn stones, stones in a higher positioned kidney, upper renal calyceal stones, complicated middle or lower renal calyceal stones) and 40 cases of ureteral stones above the level of L4. Among these 83 patients, there were 53 males and 30 females. The median age was 43 years old. There were 75 cases with hydronephrosis at varying degrees. There were 13 cases complicated with pyonephrosis. There were 5 cases who had SWL previously. There were no statistical differences in mean stone burden, age, body mass index (BMI), and preoperative hemoglobin level between the 2 groups (see Table 1).

**Table 1:** General Data Comparison

	Infracostal Access	Supracostal Access	*p* value
Number of cases number with renal stones	23	20	
Number of cases with ureteral stones	20	20	
Average stone burden (cm^2^)	7.56 ± 2.35	7.98 ± 2.29	>0.05
Percentage of staghorn stones	11/43(25.6 %)	11/40(27.5 %)	
Mean			
Age (years)	45.3 ± 15.3	42.4 ± 17.5	>0.05
BMI	25.3 ± 0.3	24.4 ± 3.2	>0.05
Mean hemoglobin level (g/dL)	12.2 ± 0.53	13.2 ± 8.2	>0.05

*BMI* Body mass index

All the patients in these 2 groups were treated successfully. No gastrointestinal damage, pleural or peritoneal effusions, pyemia, or sepsis occurred. There were 2 cases in the supracostal group that were complicated by pleural cavity injury (Clavien Grade 3). However, these 2 cases were cured by conservative therapies including closed drainage of thoracic cavity. Four cases of fever (Clavien Grade 1) were noted in each group (9 % for Group 1 and 10 % for Group 2) without significant difference. No Clavien Grade 2 or 4 complications, and other Grade 1 and 3 complications were noted in either group (see Table 2).

**Table 2:** Operative Data Comparison

		Infracostal Access	Supracostal Access	*p* value
Total number of percutaneous tracts (%)		47/43(109.3 %)	53/40(132.5 %)	0.005
Percutaneous tract (%)	Single tract	40/43(93.0 %)	29/40(72.5 %)	0.0165
	Two tracts	2/43(4.7 %)	9/40(22.5 %)	0.0209
	Three tracts	1/43(2.3 %)	2/40(5.0 %)	0.29
Average time needed for establishing a percutaneous tract (min)		(3 ± 1.6)min	(6 ± 3.9)min	0.001
Average time needed for stone clearance (min)	Renal stones	(42 ± 12)min	(61 ± 26)min	0.33
	Ureteral stones	(10 ± 6)min	(11 ± 8)min	0.29
Renal stone clearance rate by one surgery using a single percutaneous tract		18/23(78.26 %)	7/20(35.0 %)	0.002
Stone clearance rate by one surgery	Renal stones	21/23(91.3 %)	18/20(90.0 %)	0.43
	Ureteral stones	100 %	100 %	
Renal stones needing secondary treatment (secondary MPCNL or ESWL) (%)		2/23(8.7 %)	2/20(10.0 %)	0.43
Mean amount of bleeding (ml)		72.8 ± 28.1	86.74 ± 32.6	0.040
Clavien Grade 1 complication		4(9 %)	4(10 %)	0.82
Clavien Grade 2 complication		0(0 %)	0(0 %)	0.001
Clavien Grade 3 complication		0(0 %)	2(5 %)	
Clavien Grade 4 complication		0(0 %)	0(0 %)	

There was a significantly smaller total number of percutaneous tracts needed, a smaller number of cases that needed two percutaneous tracts to clear stones completely, a shorter average time in establishing a percutaneous tract, and a smaller average amount of bleeding in Group 1 (*p* < 0.05). At the same time, there was a significantly larger number of cases in which stones were cleared completely using a single percutaneous tract, and a higher renal stone clearance rate by one surgery (*p* < 0.05). However, there were no significant differences in the average renal stone clearance time, the average ureteral stone clearance time, ureteral stone clearance rate using a single percutaneous tract by one surgery, renal stone clearance rate by one surgery, and cases in which 3 percutaneous tracts were needed to clear all stones (see Table 2).

## Discussion

Currently many scholars believe that a supracostal approach to puncturing renal upper or middle calyces in order to establish the percutaneous tract can maximally break the staghorn renal and upper ureteral stones. This approach makes it easier to find the outlet of the renal pelvis and indwell a double-J pigtail stent. This belief has been based on consideration of the anatomic characteristics of the renal collecting system, because the supracostal approach is on the direction of the long axis of the renal collecting system [6, 9, 10]. In treating renal staghorn calculi using PCNL, we must first consider the safety of the surgery. A secondary consideration is to increase the stone clearance rate by one surgery. The access approach and size of the percutaneous tract, and the efficacy and flexibility of the stone breaking devices are critical factors in determining the safety and efficacy in treating staghorn renal calculi by PCNL [11].

Our patented lithotripsy and suctioning/clearance system is comprised of a patented metal sheath, a small diameter (12 F) nephroscope, and an irrigation and suctioning system (Fig. 1). Previous study indicated that the patented system group had a significantly higher percentage of stone-free outcomes after one surgery and significantly less intraoperative bleeding compared to EMS (Electro Medical System) group [7].

To perform a PCNL, we must first consider how to design the percutaneous tract access approach, in order to achieve the maximal stone clearance rate using a single percutaneous tract and reduce the injury from the establishment of multiple percutaneous tracts [9]. The percutaneous tract must maximally facilitate the movement of the sheath to clear stones and avoid complications, such as bleeding, from the movement of the sheath. Due to the interference of ribs, ultrasound image could be affected when using a supracostal approach. This will affect the accuracy of puncturing. In the meantime, the large number of blood vessels in the upper and middle poles of the kidney will increase the risk of puncturing. In addition, there is potential risk of pleural and lung injury when using a supracostal approach [12, 13]. In our study, we did see this complication in 5 % of our cases in the supracostal group, and none in the infracostal group. Also, by using the supracostal access approach, the narrow intercostal space limited the movement of the sheath. This can affect stone clearance. In our study, we found that there was higher need for multiple percutaneous tracts for stone clearance in the supracostal group. We also found stone clearance to be more efficient when a second or/and third percutaneous tract was/were established using an infracostal approach. In our supracostal group, there were 13 percutaneous tracts established using an infracostal approach. The higher the number of percutaneous tracts needed, the higher the risk of bleeding. Because there are fewer blood vessels in the rear part of the lower renal pole, the risk of serious bleeding from puncturing is lower when using an infracostal approach. The ultrasound images are clearer when using the infracostal approach because there is no interference from the ribs. This makes the puncturing more convenient and more accurate [14]. Our study revealed that there were significant differences in the total number of percutaneous tracts, ratio of cases needing a single percutaneous tract, ratio of cases needing 2 percutaneous tracts, average time in establishing a percutaneous tract, and average amount of bleeding. This indicated the safety and practicability of the infracostal access approach.

**Fig. 2:**
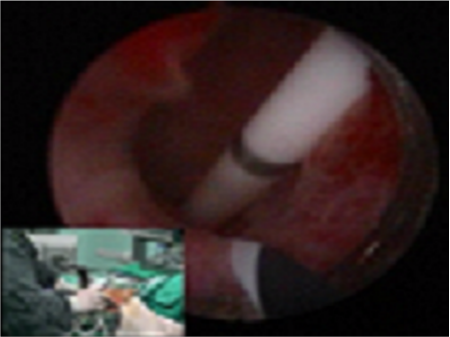
The outlet of the renal pelvis and upper ureter can be visualized clearly using an infracostal approach

**Fig. 3:**
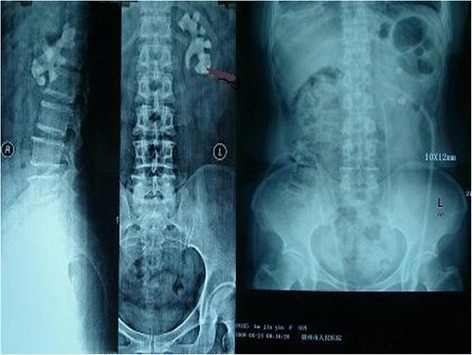
Comparison of preoperative and postoperative KUBs of a patient with left renal staghorn stone (*Red line* represents the direction of infracostal access approach; *Left*, Preoperative KUB; *Right*, Postoperative KUB)

**Fig. 4:**
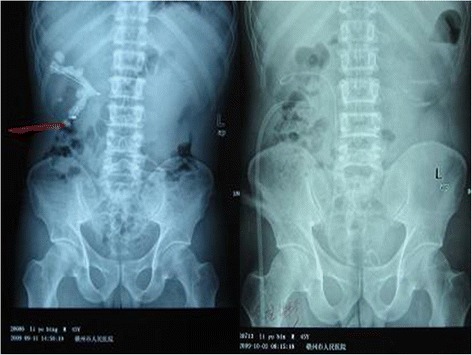
Comparison of preoperative and postoperative KUBs of a patient with right renal multiple stones (*Red line* represents the direction of infracostal access approach; *Left*, Preoperative KUB; *Right*, Postoperative KUB)

**Fig. 5:**
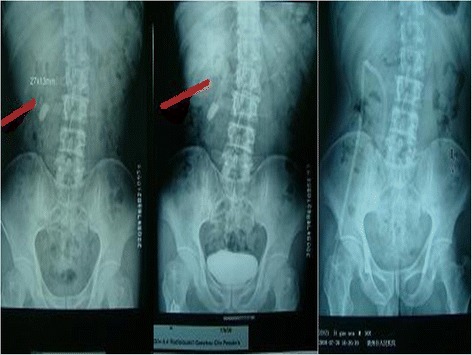
Comparison of preoperative and postoperative images of a patient with right upper ureteral stone (*Red line* represents the direction of infracostal access approach; *Left*, Preoperative KUB; *Middle*, Preoperative IVU; *Right*, Postoperative KUB)

Regardless of the approach was used, the principle is to extract stones along the long axis of the kidney. Many urologists have concerns that when using the infracostal access approach it becomes difficult for the scope and sheath to access the outlet of the renal pelvis and the upper segment of the ureter, therefore affecting the indwelling of a double-J pigtail stent or stone clearance of upper ureteral stones. However, the material of our patented sheath is hard, not easily deformed, and small in diameter, maximizing access. For each surgery in the infracostal group, we did not booster the abdomen to immobilize the kidney. The increased ability of the kidney and ureter to move facilitates the hard sheath's access to all target locations. Because the patented sheath is small, and its range of movement is increased, it can easily access most of the renal calyces and the upper ureter in order to explore and remove stones under direct vision, reducing the number of percutaneous tracts required for multiple or staghorn kidney stones, thereby reducing kidney damage. Using the patented system to clear stones in patients with significant hydronephrosis, we can suck away the hydrops and reduce the volume of the kidney easily. Thus the perirenal space is increased, the tension in the renal pelvis is decreased, and the movement of the kidneys and ureter is therefore increased. This will further facilitate access by the sheath to different areas in order to break and remove the stones. For some staghorn stones with a wide angle, we usually broke the part in the renal pelvis first, in order to create space. Then we moved the stones located in the small calyces using the hard sheath to the renal pelvis under a video monitor to do lithotripsy. For secondary stones impacted inside small calyceal necks, we usually dilated the calyceal neck first, then did lithotripsy inside the calyces or broke and/or sucked stones by putting the sheath immediately outside the calyceal necks. For slush like stone materials, we used the sheath to suck them away quickly. Due the larger movements of the scope, kidney stone clearance rate by one surgery was significantly higher in the infracostal group (Fig. 2, 3, 4, 5).

## Conclusions

There were several advantages of infracostal access in treating renal stones and upper ureteral stones. These include accuracy in establishing a percutaneous tract, safety, quickness, convenience and flexibility in moving the patented sheath, and higher renal and upper ureteral stone clearance rate by one surgery. However, we do recognize that our sample size was not large. Larger, prospective studies in multiple clinical centers are warranted to further compare the supracostal and infracostal access approaches in treating upper urinary tract stones using MPCNL with the aid of our patented system.

## Abbreviations

MPCNL: Minimally invasive percutaneous nephrolithotomy
PCNL: Percutaneous nephrolithotomy
SWL: Shock wave lithotripsy
KUB: Plain film of kidney-ureter-bladder
CT: Computerized tomography
EMS: Electro Medical System
